# Supplementary material for: Content-rich biological network constructed by mining PubMed abstracts
Source: BMC Bioinformatics. 2004 Oct 8;5:147. doi: 10.1186/1471-2105-5-147 (PMC528731; doi:10.1186/1471-2105-5-147)
Supplement: Additional File 5 — The original Chilibot query results of the term "long-term potentiation (LTP)" and 22 other terms, limiting the latest references analyzed to the years 1990, 1995, 2000, and 2004. [file 1471-2105-5-147-S5.bz2 › chilibotAdditionalFile5/ltp2000/html/LTP_AMPA.html]

 


 **LTP** and **AMPA** 
  
Found 215 abstracts in PubMed,  **30 abstracts were retrieved and analyzed**.  


---

 Search Google  |
 PDF files only 
|  EDU domain only 

---

**Interactive relationship** (e.g. stimulation, inhibition, etc)

- The mechanism is based on a postulate that phosphorylation dephosphorylation of  **AMPA**  GABAA receptors on cerebellar cells causes the  **LTP**  LTD of excitatory LTD  **LTP**  of inhibitory transmission.  Ref: 10923375 Zh Vyssh Nerv Deiat Im I P PavlovaZh Vyssh Nerv Deiat Im I P Pavlova, 2000
- Thus, at pharmacologically relevant concentrations these  **AMPA**  receptor antagonists apparently do not suppress  **LTP** , a cellular mechanism underlying memory formation.  Ref: 10974490 Brain Res Bull, 2000
- The induction of long term potentiation  [ **LTP** ]  again produced a decrease in the size of responses to NMDA and  **AMPA** .  Ref: 10862824 Eur J Pharmacol, 2000
- **AMPA**  receptor antagonists, GYKI 52466 and NBQX, do not block the induction of long term potentiation  [ **LTP** ]  at therapeutically relevant concentrations.  Ref: 10974490 Brain Res Bull, 2000
- For example, certain  **AMPA**  modulators facilitate long term potentiation  [ **LTP** ] , which is considered a cellular mechanism that may be important for memory storage and they also facilitate memory encoding in behavioural experiments.  Ref: 11060708 Expert Opin Investig Drugs, 2000
- Long term potentiation  [ **LTP** ]   **LTP**  or increased activity of the calcium calmodulin dependent protein kinase II CaMKII induced delivery of tagged  **AMPA**  Rs into synapses.  Ref: 10731148 Science, 2000
- Induction of  **LTP**  or LTD altered the short term plasticity of transmission onto both pyramidal cells and interneuron CP  **AMPA**  synapses by a mechanism consistent with changes in release probability.  Ref: 11069934 J Neurosci, 2000
- However, the induction of long term potentiation  [ **LTP** ]  produced a marked loss of sensitivity to quinolinic acid, with smaller effects on NMDA,  **AMPA**  and kainate responses.  Ref: 10862824 Eur J Pharmacol, 2000
- The induction of graded burst discharges in magnesium free conditions and the expression of burst discharges evoked in normal bathing medium with subsequent disinhibition were both blocked by DL 2 amino 4 phosphonovaleric acid APV and were therefore NMDA receptor dependent, in contrast to long term potentiation  [ **LTP** ]   **LTP**  in the perforant path, which is induced by NMDA receptors and is also expressed by alpha amino 3 hydroxy 5 methyl 4 isoxazoleproprionate  **AMPA**  receptors.  Ref: 11110816 J Neurophysiol, 2000
- These results show that  **LTP**  and CaMKII activity drive  **AMPA**  Rs to synapses by a mechanism that requires the association between GluR1 and a PDZ domain protein.  Ref: 10731148 Science, 2000
- Here we show that  **LTP**  and LTD reversibly modify the phosphorylation of the  **AMPA**  receptor GluR1 subunit.  Ref: 10879537 Nature, 2000
- Driving  **AMPA**  receptors into synapses by  **LTP**  and CaMKII requirement for GluR1 and PDZ domain interaction.  Ref: 10731148 Science, 2000
- **LTP**  and LTD may be mediated by the modulation of  **AMPA**  alpha amino 3 hydroxy 5 methyl 4 isoxazloe proprionic acid receptor phosphorylation.  Ref: 10879537 Nature, 2000
- **LTP**  converts silent synapses to functional ones by causing the rapid appearance of  **AMPA**  currents.  Ref: 10504196 J Neurobiol, 1999
- Both alpha amino 3 hydroxy 5 methyl 4 isoxazole propionate  **AMPA**  and N methyl D aspartate NMDA glutamatergic receptor subtypes in hippocampus have been shown to express long term potentiation  [ **LTP** ]   **LTP** , a form of synaptic modification believed to be involved in memory formation.  Ref: 10456082 Learn MemLearn Mem, 1999
- According to several lines of experimental evidence, changes in synaptic function observed with  **LTP**  and LTD are thought to be the result of modifications of postsynaptic currents mediated by the alpha amino 3 hydroxy 5 methyl 4 isoxazole propionate  **AMPA**  subtype of glutamate receptors.  Ref: 11092449 Cell Mol Life Sci, 2000

**Parallel relationship** (e.g. studied together, co-existance, homology, etc.)

- The involvement of alpha amino 3 hydroxy 5 methylizoxazole 4 propionic acid  **AMPA**  receptors in induction of long term potentiation  [ **LTP** ]   **LTP**  was examined in rat hippocampal slice preparation.  Ref: 10974490 Brain Res Bull, 2000
- Finally, we will review data relevant to the issue of selective changes in  **AMPA**  binding after environmental enrichment and  **LTP** .  Ref: 11092449 Cell Mol Life Sci, 2000
- The unitary postsynaptic mechanism of plasticity in striatum, neocortex, hippocampus and cerebellum involves the  **LTP**  LTD excitation as result of  **AMPA**  and NMDA receptor phosphorylation dephosphorylation, while the  **LTP**  LTD of inhibition is the result of the GABA receptor phosphorylation dephosphorylation.  Ref: 10885014 Ross Fiziol Zh Im I M Sechenova, 2000
- Thus, NT 3 produces synapse specific and age dependent  **LTP**  like enhancement of  **AMPA**  kainate receptor mediated synaptic transmission in the spinal cord, and this action requires the availability of functional NMDA receptors in the motoneuron.  Ref: 10938302 J Neurophysiol, 2000
- this signaling mechanism is distinct from  **LTP**   **AMPA** .  Ref: 10454358 Learn MemLearn Mem, 2000
- NT 3 evokes an  **LTP**  like facilitation of  **AMPA**  kainate receptor mediated synaptic transmission in the neonatal rat spinal cord.  Ref: 10938302 J Neurophysiol, 2000
- Long term potentiation  [ **LTP** ]   **LTP**  of alpha amino 3 hydroxy 5 methyl 4 isoxazolepropionic acid  **AMPA**  and N methyl D aspartate NMDA receptor mediated components of dual component field excitatory postsynaptic potentials fEPSP A and fEPSP N was studied in the CA1 stratum radiatum in hippocampal slices of rats.  Ref: 10825494 Brain Res, 2000
- Inhibition of nitric oxide NO synthase prevented the long term potentiation  [ **LTP** ]  induced loss of responsiveness to NMDA... implying a role for NO in the loss of NMDA sensitivity.  Ref: 10862824 Eur J Pharmacol, 2000
- It is based on the assumption that, in diverse central nervous system structures, long term potentiation  [ **LTP** ]  depression  **LTP**  LTD of excitatory transmission depression potentiation of inhibitory transmission, LTDi LTPi is the result of an increasing decreasing the number of phosphorylated  **AMPA**  and NMDA GABA A receptors.  Ref: 11084240 BiosystemsBiosystems, 2000
- Although STP for both receptor subtypes decayed to an asymptotic, steady state level of  **LTP**  and could be induced repetitively, there were substantial differences in several aspects of  **AMPA**  and NMDA STP dynamics.  Ref: 10456082 Learn MemLearn Mem, 2000
- S dihydro cyclopentano, 4 benzothiadiazine dioxi de S 18986 1 is a new compound that facilitates post synaptic responses by modulating alpha amino 3 hydroxy 5 methyl 4 isoxazole propionic acid  **AMPA**  receptor mediated synaptic responses and thus promotes long term potentiation  [ **LTP** ]  and potentiates S  **AMPA**  induced release of noradrenaline in rat brain slices.  Ref: 10924928 Eur J Pharmacol, 2000
- Single pulse stimulation of perforant path afferents paired with postsynaptic depolarization also induced  **LTP**  of both  **AMPA**  and NMDA components.  Ref: 10456082 Learn MemLearn Mem, 2000
- Because anandamide does inhibit long term potentiation  [ **LTP** ] , and cannabinoid abuse is known to affect learning and memory, the effects of anandamide on recombinant  **AMPA**  glutamate receptor GluR subunit currents were studied in Xenopus oocytes.  Ref: 10543424 Naunyn Schmiedebergs Arch Pharmacol, 1999
- High frequency stimulation of mossy fibers induced long term potentiation  [ **LTP** ]   **LTP** , long term depression LTD, or no change at pyramidal neuron synapses, interneuron CP  **AMPA**  synapses, and CI  **AMPA**  synapses, respectively.  Ref: 11069934 J Neurosci, 2000
- In the present study we have examined the role of PKC in tetanus induced  **LTP**  of  **AMPA**  receptor mediated synaptic transmission in hippocampal slices obtained from adult rats.  Ref: 11069602 Eur J Neurosci, 2000
- Silent synapses show responses from high affinity NMDA receptors NMDARs but not low affinity  **AMPA**  receptors AMPARs, but gain AMPAR responses upon long term potentiation  [ **LTP** ]   **LTP** .  Ref: 10725921 Nat Neurosci, 2000
- The mechanisms underlying the differential expression of long term potentiation  [ **LTP** ]   **LTP**  by  **AMPA**  and NMDA receptors, are unknown, but could involve G protein linked metabotropic glutamate receptors.  Ref: 10454358 Learn MemLearn Mem, 2000
- In this study, electrophysiological recordings from dentate granule cells were used to compare the potentiation of  **AMPA**  and NMDA receptor mediated responses occurring during the initial phase of  **LTP** , typically identified as STP.  Ref: 10456082 Learn MemLearn Mem, 2000
- In contrast, CA1 neurons from mGluR5 deficient mice showed a complete loss of the NMDA receptor mediated component of  **LTP**   **LTP**  NMDA, but normal  **LTP**  of the  **AMPA**  receptor mediated component  **LTP**   **AMPA** .  Ref: 10454358 Learn MemLearn Mem, 2000
